# Dynamic Modeling and Simulation Analysis of an Arm Rehabilitation Robot With Mobile Platform

**DOI:** 10.1155/abb/8254911

**Published:** 2025-05-27

**Authors:** Tao Gong, Yuanyuan Lin, Yufeng Wang, Wenbin Wang, Wei Chen, Jiancheng (Charles) Ji

**Affiliations:** ^1^Institute of Intelligent Manufacturing, Shenzhen Polytechnic University, 4089 Shahe West Road, Shenzhen 518055, China; ^2^Medical Insurance Department, Weifang Hospital of Traditional Chinese Medicine, 1055 Weizhou Road, Weifang 261042, China; ^3^Rehabilitation Medicine Department, People's Hospital of Gaoxin, 768 Fudong Road, Weifang 261000, China

## Abstract

In response to the ever-increasing demand of upper limb rehabilitation training and function improvement, a novel arm rehabilitation robot with mobile platform, which can move on the table, is designed to facilitate the upper limbs strength training via the passive force field. The proposed robot provides three passive degrees of freedom (plane motion on the table and rotation around the vertical axis), which can fulfill the robot-aided trajectory training for upper limbs, such as shoulder, elbow, and wrist flexion/extension. Meanwhile, changed force field was established on the table via three elastic ropes. The user first connects the robot by the grab handle and arm support, then the screen displays the reference trajectory (RT) and notifies the user to manipulate the robot to draw the trajectory, the actual trajectory is calculated via the sensors and displayed to feed the user to adjust the muscular strength. In this paper, the reference trajectories, corresponding angular velocity of the wheels and the distribution of force field are analyzed via the dynamic modeling. Simulation studies are carried out to analyze the effectiveness of the theoretical model, kinematic performance, and distribution of force field. The path tracking simulation results showed that the peak error was 1.2 mm for the “∞” curve and 0.9 mm for the “O” curve. The force analysis results showed that the robot can generate the circinate force field and the maximum force of the whole workspace was 9.8 N with the elastic rigidity was 20 N/m. Furthermore, the maximum force was 11.02 N and the minimum force was 6.48 N when the robot moved along the of the circular path. These results demonstrate that the robot can generate appropriate force field to facilitate the motor training and paved the way to interventional therapy of the robot in the future. We will optimize the structure parameters and assemble a prototype to test the performance of the robot.

## 1. Introduction

In 2019, there were 12.2 million incident cases of stroke and 101 million prevalent cases of stroke [1], a leading cause of long-term disability [2]. About 85% of stroke victims experience arm weakness [3] and only 20%–56% of them recover part or whole motor function [4]. Besides, neurological impairment after stroke can lead to the muscle rigidity and joint adhesion, causing the lack of exercise [5]. The main drawback of the conventional treatments is not able to meet the needs of intensively or frequently as necessary for the cost and availability of therapists [6]. To solve above problems, various rehabilitation robots are developed to implement the task-specific practice with a large number of repetitions [7–9]. However, large upper limb exoskeletons used in clinics are prohibitively expensive and passive training equipment and simple rehabilitation robots are more affordable for most patients to recover motor functions [10–12].

To date, different types of rehabilitation robots have been developed to enhance arm motor recovery by restoring the arm motions [13, 14]. The conventional robots guide the arm to finish joint motions and replace the therapists to implement the repeatability rehab training, such as the MIME and the ARM Guide robot [15]. With the development of robot technology, robotic assisted upper extremity repetitive therapy with electronic components have emerged to provide function training and better assistance [16]. One of the successful robots is the MIT-MANUS from Massachusetts Institute of Technology [17], this robot is able to control the motion trajectory via impedance adjustment. Many studies have addressed this type of robot because it demonstrates a promising way to enhance the motor function of upper limbs. For instance, Melendez-Calderon et al. [18] developed a robotic trainer to provide sport training and rehabilitation by providing visual information about the position error. Furukawa et al. [19] developed an upper extremity exoskeleton robot and studied the electromyography (EMG)-based assistive robot control method. But with the development of neurosciences, the traditional method and theory were found that the reiterative training may be not an optimal way to facilitate the motor relearning. Li et al. [20] proposed a cable-driven lower limb exosuit and studied the human-in-the-loop control method via impedance learning [21]. A systematic review from the Huazhong University of Science and Technology demonstrated that robot-assisted training was slightly superior in motor impairment recovery [22]. Furthermore, Lum et al. [23] compared the robot-assisted training with the conventional therapy techniques for the upper-limb motor function rehab, and the literatures proposed the important robot-assisted and the EMG arm training methods for upper-limb motor function rehab, which promoted the development of the robot-assisted training technology [24–27].

At present, simple and low-cost robotic devices have been rarely reported in stroke patients [28]. The passive robotic system (ArmAssist) from University of Cordoba has the functions of physical support, interactive games operating on a web-based platform [29]. A novel shoulder exoskeleton robot with parallel actuation and passive slip interface has been proposed by Hunt et al. [30] for rehabilitation or augmentation of the human shoulder. However, low-cost robotic devices were discussed in the literature for the low and middle income countries [31], such as the Haptic Theradrive, the HERO Grip Glove and the CAAR robot, but the robotic devices with force field and position feedback remains to further study. To assist table-based rehabilitation with a simple structure, we propose to design an arm rehab robot with omni-directional mobile platform (OMP) for upper limb rehabilitation. The advantages of this design are given below:

a. To satisfy planar motion of the arm, assist the user in reaching training with assistant force and arm-support.
b. To design a feedback system based on the dynamic modeling and force sensors, three elastic ropes were used to generate a force field in the table.
c. To establish the adjustable force field through various spring stiffness and provide challenge, enjoyment, and assessment for patients to facilitate the motor learning.

The rest of the paper is divided into five sections. In Section 2, the training method of the robot is introduced and the conceptual design is detailed. The kinematics and dynamics of the OMP are studied in Section 3. Performance analysis such as workspace, motion trajectory, and force field analysis is conducted in Section 4. Finally, conclusions and future work are given in Section 5.

## 2. System Description

According to statistics of the upper limb rehabilitation robot report, the global upper limb rehabilitation robot market sales reached 4.4 billion in 2023 and the market increased in size by 34%. The market requirements come from the ever-increasing demands of stroke patients with the upper limb disorder. The design requirements originate from the user requirements analysis (URA) and advantages–disadvantages analysis of existing robots, the patients with upper limb movement disorders after stroke need upper limbs strength training and joint motions. The most used classification method classified the upper limb rehabilitation robots into two categories: the end effector and the exoskeleton. The end effector is attached to the human arm to pull the human upper limb for rehabilitation training and the rehabilitation training mode of the patient is adjusted according to the robot's end motion planning.

Our research is focused on the realization of upper limbs strength training with an OMP, which is designed to offer an assistive force and position feedback for a patient during the training. The arm rehabilitation robot consists of an OMP, a position detecting system (PDS) and an interactive software system (ISS). The mobile platform has three passive omni wheels to achieve the planar motion on the table. The PDS has three elastic ropes that provides assistive force and three tension sensors to detect the position and pose of the robot. The ISS has a screen to reveal the center of robot.

### 2.1. System Description

**Figure 1:**
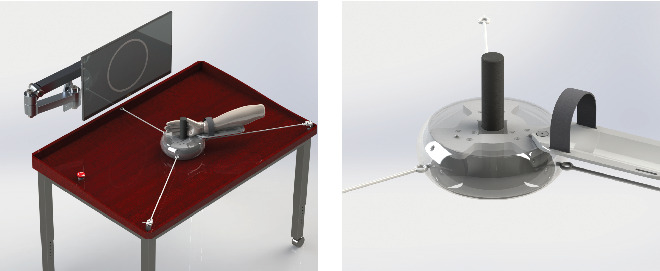
Conceptualized design of the arm rehabilitation robot. Three sensors are fixed on the table and three elastic ropes were used to connect the robot with the sensors.

Figure 1 shows the design model of the arm rehabilitation robot for upper limbs strength training. The robot consists of three main parts: (i) an OMP; (ii) a PDS and (iii) an ISS. The purpose of the OMP is to provide planar motion on the table, thereby, the robot can assist the training of the shoulder, elbow, and wrist joints. It is designed as a curling-shaped frame to provide the patient with a free space of 0.6 m in the lateral direction and a free space of 0.5 m in the longitudinal direction on the table. The OMP is supported with three passive omni wheels which are uniformly distributed under the circular frame. It is advantageous to improve maneuverability of the robot by installing the three omni wheels. By contrast, most existing devices implement the plane motions by two actuating motors with connecting rods or linear motion equipment, and the PLC/PC motor actuators are necessary for the control system. That is one of the reasons that the proposed robot is more affordable for most patients.

The purpose of the PDS is to provide assistive force in the plane during training and detect the position and pose of the robot. It is designed to have three elastic ropes and three tension sensors (S-shaped tension sensor, SBT630-50N, SIMBATOUCH Co., Ltd.), the sensors are installed on the table, and the sensors are uniformly distributed on a circle. The elastic ropes are used to connect the robot and three tension sensors respectively, besides, the elastic ropes are pretensioned to ensure the tension of rope. The calculative process of the monitoring of the position and pose will discuss in the Section 3. The ISS is designed to be installed on the front of the table to reveal the center of robot with a screen and the control cabinet and sensor amplifiers are installed under the table.

In summary, to implement the upper limb motor training, the robot connects the user's arm by an adjustable forearm and grab handle first and then the screen displays the reference trajectory (RT) and real-time position of the robot center, the real-time position, and pose of the robot can be obtained by the kinematics of the three elastic ropes. Through the sensor amplifier and filter, the minicomputer fixed under the table can calculate the position and pose of the robot. The elastic ropes can establish a two-dimensional force field on the table and be used to confirm the absolute location of the robot, which may enhance the patient's strength training intensity.

### 2.2. Training Method

The training strategy of the robot is to assist patients with arm offloading and natural arm movements. For this purpose, an OMP is designed to support the arm weight and then the user can manipulate the robot to finish the training games displayed on the screen. The training games include daily tasks that require user's active participation to motivate the initiative and enhance the effectiveness.

**Figure 2:**
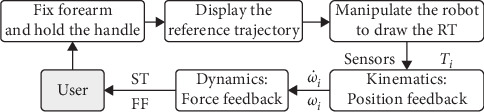
Training architecture of the arm rehabilitation robot.

The training architecture of the robot is shown in Figure 2, the first step is to fix the forearm and hand on the robot, then the screen displays the RT (pink full line), and real-time position of the robot center (black dot), the reference trajectories include complex tasks that require variable cognitive engagement. The user is informed to draw the RT with minimum error as best as they can and the sensors obtain the tension of the elastic ropes **T**_*i*_. With the help of the kinematics and dynamics of the robot, the minicomputer calculates the real-time position and pose of the robot, angular velocity, and angular acceleration of each wheel. From this, the simulated trajectory (ST; black center line) and feedback force can be calculated and provided. The simulated trajectories and force field will be tested in the Section 4.

## 3. Dynamic Modeling

To provide patients with absolute location and force feedback, the kinematic modeling and dynamic modeling of the rehabilitation robot are derived in this section. In kinematic modeling, the rehabilitation robot is divided into two parts: the mobile platform and the elastic ropes, and the vectorial method [29, 32] is used for kinematic modeling in this paper. The robot is capable of the plane motion on the table and rotation around the vertical axis; to detect the position and pose of the robot, we use three elastic ropes to connect the fixed sensors and the moving robot, on the premise of without loose ropes, we can obtain the position and pose of the robot via the length of the three ropes. And the length of the three ropes can be calculate by the tension via the sensors, so, we can obtain the position and pose of the robot by the sensor readings.

### 3.1. Kinematics of the Robot

**Figure 3:**
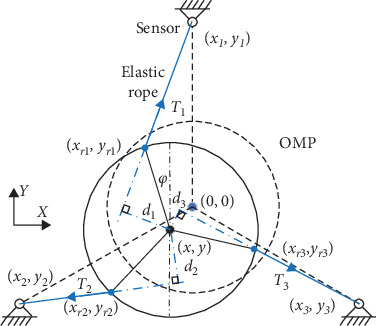
Schematic diagram of the robot. The OMP represents the omni-directional mobile platform. The dotted line represents the initial position (0, 0) and pose (**φ** = **0**) of the robot with the blue center and the solid line represents the current position and pose of the robot with the black center. The blue lines represent the three elastic ropes and the fixed symbols represent the three sensors.

The kinematic model of the arm rehabilitation robot is presented in Figure 3 and the *OXY* is set as a global coordinate system with the origin at a point in space. *o*_0_*x*_0_*y*_0_ is the local coordinate system attached to the OMP with *o*_0_ at the center of the robot frame and its position and orientation is defined as $p_{R}=[\begin{array}{cc} x & y \end{array}{]}^{T}$ and **o**_R_ = *φ*, where *x* and *y* denote the position of the center of the OMP and *φ* denotes the heading angle of the OMP about the *Z*-axis. The initial position is $\left(\begin{array}{c} 0,0 \end{array}\right)$ and initial orientation is 0, $\left(\begin{array}{c} x_{i},y_{i} \end{array}\right)\left(i=1,2,3\right)$ denote the position coordinates of the fixed sensers. $\left(\begin{array}{c} x_{ri},y_{ri} \end{array}\right)\left(i=1,2,3\right)$ represent the position coordinates of the installation of elastic ropes on the robot, *i* represent the number of the wheel. The *l*_0*i*_(*i* = 1, 2, 3) and *l*_*i*_(*i* = 1, 2, 3) denote the initial length and current length of the elastic ropes respectively, and *d*_*i*_(*i* = 1, 2, 3) represent the distance from the *o*_0_ to the vector of elastic ropes. The radius of the robot frame is *R* and the radius of the omni-wheel is *r*.

The first part of the kinematics is to calculated the position and orientation of the robot from the three tension sensors, radius of the robot frame is *R*, the $\left(\begin{array}{c} x_{ri},y_{ri} \end{array}\right)\left(i=1,2,3\right)$ can be expressed as:

(1) $$\begin{array}{c} \left\{\begin{array}{c} x_{r1}=x-R\;\sin\varphi \\ y_{r1}=y+R\;\cos\varphi \end{array}\right.. \end{array}$$

(2) $$\begin{array}{c} \left\{\begin{array}{c} x_{r2}=x-R\;\sin\left(\pi /3-\varphi \right)\\ y_{r2}=y-R\;\cos\left(\pi /3-\varphi \right) \end{array}\right.. \end{array}$$

(3) $$\begin{array}{c} \left\{\begin{array}{c} x_{r3}=x+R\;\sin\left(\pi /3+\varphi \right)\\ y_{r3}=y-R\;\cos\left(\pi /3+\varphi \right) \end{array}\right.. \end{array}$$

Then, under the assumption of the elastic ropes without loose state, the current length of the elastic ropes *l*_*i*_(*i* = 1, 2, 3) can be expressed by the distance from position coordinates of the fixed sensers to the installation location of the elastic ropes:

(4) $$\begin{array}{c} l_{i}=\sqrt{{\left(x_{ri}-x_{i}\right)}^{2}+{\left(y_{ri}-y_{i}\right)}^{2}}, \end{array}$$

and its intersection angles of each rope *θ*_*i*_(*i* = 1, 2, 3) can be obtaioned:

(5) $$\begin{array}{c} \theta_{i}=\cos^{-1}\left(\frac{\left(x_{ri}-x_{i},y_{ri}-y_{i}\right)\cdot \vec{x}}{\left|\left(x_{ri}-x_{i},y_{ri}-y_{i}\right)\right|\cdot \left|\vec{x}\right|}\right). \end{array}$$

Assuming that the vector of *d*_*i*_(*i* = 1, 2, 3) is (*a*, *b*), then, the vector of the tension can be expressed as:

(6) $$\begin{array}{c} {\vec{n}}_{i}=\frac{\left(x_{ri}-x_{i},y_{ri}-y_{i}\right)}{\left|x_{ri}-x_{i},y_{ri}-y_{i}\right|}=\left(-b,a\right). \end{array}$$

So, the arm of tension force (distance from the *o*_0_ to the vector of elastic ropes) can be obtained:

(7) $$\begin{array}{c} \left|d_{i}\right|=\frac{\left(x_{ri}-x_{i},y_{ri}-y_{i}\right)\cdot {\vec{d}}_{i}}{\left|{\vec{d}}_{i}\right|}. \end{array}$$

For the second part of the kinematics, the twist of the OMP in the robot frame is expressed as $t_{R}=[\begin{array}{ccc} {\dot{x}}_{R} & {\dot{y}}_{R} & {\dot{\varphi }}_{R} \end{array}{]}^{T}$, and the rotation speeds of the three omni-wheels are defined as $\omega_{w}=[\begin{array}{cc} {\dot{q}}_{1} & \begin{array}{cc} {\dot{q}}_{2} & {\dot{q}}_{3} \end{array} \end{array}{]}^{T}$, subscript l, 2, and 3 indicating front, left, and right wheel, respectively. Under the assumption of pure rolling without slippage, the kinematic equation of the OMP can be expressed:

(8) $$\begin{array}{c} \omega_{w}=J^{-1}t_{M}, \end{array}$$

where

(9) $$\begin{array}{c} J^{-1}=\frac{1}{r}\left[\begin{array}{ccc} -1 & 0 & R\;\cos\left(-\pi /6\right)\\ 0.5 & -0.866 & R\;\cos\left(-\pi /2\right)\\ 0.5 & 0.866 & R\;\cos\left(5\pi /6\right) \end{array}\right]. \end{array}$$

By multiplying the transformation matrix, the twist of the OMP in the global frame $t_{G}=[\begin{array}{ccc} \dot{x} & \dot{y} & \dot{\varphi } \end{array}{]}^{T}$ is expressed as:

(10) $$\begin{array}{c} t_{G}=R_{T}t_{R}. \end{array}$$

### 3.2. Dynamics of the Robot

**Figure 4:**
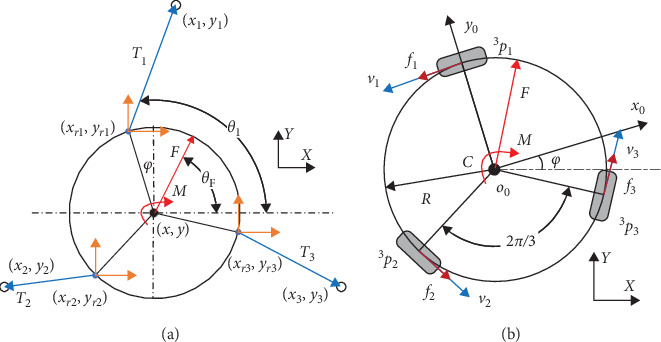
Forces and moments on the omni-directional mobile platform. ^**3**^***p***_***i***_(***i*** = **1**, **2**, **3**) represents the three omni-wheels. (a) Tension of ropes. (b) Inertia and friction force.

As mentioned previously, the rehab robot addressed on the assistance force for the upper limbs strength training. To calculate the force field with respect to the workspace on the table, as shown in Figure 4, a backward recursive method [33] is employed to derive the forces and moments acting on the end-effector as:

(11) $$\begin{array}{c} w_{h}=M_{o}{\dot{t}}_{G}+B_{o}+H_{e}w_{e}, \end{array}$$

where **w**_*h*_ is the wrench including force vector and moment vector acting on the user's hand, **M**_*o*_ is the generalized mass matrix of the robot, **B**_*o*_ is the matrix including centrifugal forces and gyroscopic moments, **H**_*e*_ is the transformation matrix, and **w**_*e*_ is the wrench of external force and moment.

For calculating the force vector **F**, the equation of forces can be written as:

(12) $$\begin{array}{c} F=m\dot{v}+\sum_{i}^{n=3}T_{i}+\sum_{i}^{n=3}f_{i}, \end{array}$$

where $\dot{v}=[\begin{array}{cc} \ddot{x} & \ddot{y} \end{array}{]}^{T}$ is the accelerated velocity of the robot, $f_{i}=\left(I{\ddot{q}}_{i}+{\dot{q}}_{i}I{\dot{q}}_{i}\right)/r$ is the force of friction, and *m* is the mass of the robot. The external force generated by the elastic ropes can be expressed as:

(13) $$\begin{array}{c} T_{i}=k_{i}\Delta_{i}=k_{i}\left(l_{i}-l_{0i}\right). \end{array}$$

*k* represents the elastic coefficient and ***I*** represents the rotational inertia and the friction force generated by the omni-wheels can be expressed as:

(14) $$\begin{array}{c} f_{i}=\left(I{\ddot{q}}_{i}+{\dot{q}}_{i}I{\dot{q}}_{i}\right)/r. \end{array}$$

## 4. Performance Analysis

Based on the above dynamic modeling, a capability map of the robot can be obtained through reachability analysis and the changing curve of the elastic ropes can be presented. It is shown that once the rope stiffness is defined, the assistance force within the workspace can be calculated via the dynamic model.

### 4.1. Kinematic Analysis

In the first simulation study, a commonly used “∞” curve was painted on the screen as the RT. For tracking error analysis, the target “∞” path was expressed as:

(15) $$\begin{array}{c} x_{r}\left(n\right)=A\frac{\cos\left(n\right)}{\sin^{2}\left(n\right)+1}, \end{array}$$

(16) $$\begin{array}{c} y_{r}\left(n\right)=B\frac{\sin\left(n\right)\cos\left(n\right)}{\sin^{2}\left(n\right)+1}, \end{array}$$

where *A* and *B* were the constants defining the length in *X*-axis and Y-axis, respectively, *n* is the number of the point. And a circular curve was painted as the RT in the second simulation study, which was expressed as:

(17) $$\begin{array}{c} x_{r}\left(n\right)=r_{o}\cos\left(2\pi n/N\right), \end{array}$$

(18) $$\begin{array}{c} y_{r}\left(n\right)=r_{o}\sin\left(2\pi n/N\right), \end{array}$$

where *r*_*o*_ was the radius of the RT and *N* is the maximum of *n*. The actual position and orientation of the robot were calculated by the three sensors. First, the twist of the MP in the global frame with Equation (5) was updated in relation time *t* step by the following equations:

(19) $$\begin{array}{c} \left\{\begin{array}{c} \theta_{n}=\theta_{n-1}+\dot{\theta }\\ x_{n}=x_{n-1}+\sin\left(\theta_{n}\right)\left|v\right|\\ y_{n}=y_{n-1}+\cos\left(\theta_{n}\right)\left|v\right| \end{array}\right.. \end{array}$$

**Figure 5:**
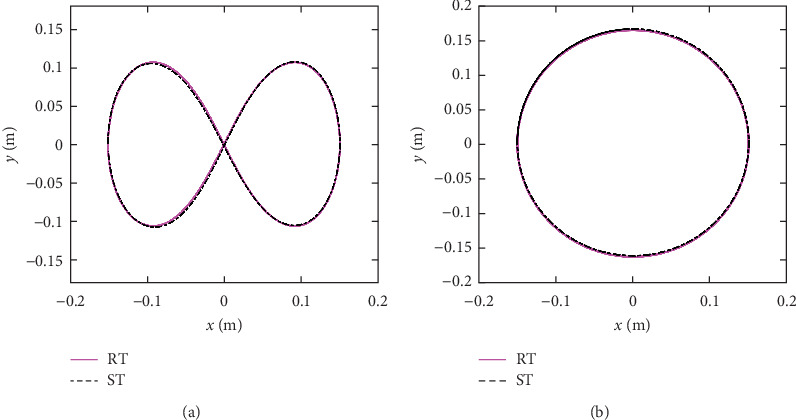
The reference trajectory (RT, pink full line) and simulated trajectory (ST, black center line). (a) “∞” path. (b) Circular path.

By calculating the error between the actual position (*x*_*n*_, *y*_*n*_) and reference position samples (*x*_*r*_(*n*), *y*_*r*_(*n*)), the normalized integral square error cost function was used to evaluate the path tracking error.  Figure 5 shows the path tracking measurement results, the pink full line represents the RT and the black center line represents the ST. According to the results, for the “∞” curve, the peak error was 1.2 mm and mean error was 0.11 mm; for the circular curve, the peak error was 0.9 mm and mean error was 0.04 mm.

**Figure 6:**
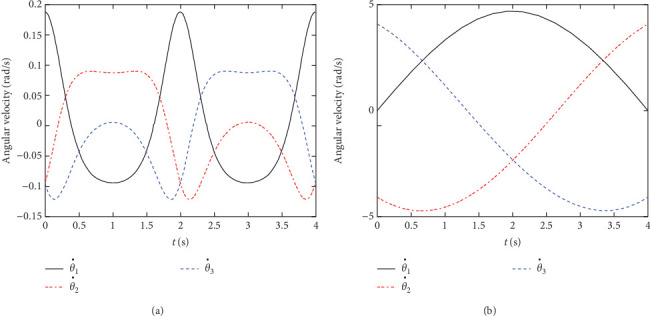
Angular velocity of the three omni-wheels during the training games. (a) “∞” path. (b) Circular path.

Figure 6 shows the angular velocity results for the two simulation studies. The Figure 6a shows the changing curve of the three omni-wheels during the “∞” path tracking game and the Figure 6b shows the changing curve of the three omni-wheels during the circular path tracking game. Through above analysis, we can draw a conclusion that the simulated trajectories are essentially coincident with the reference trajectories.

### 4.2. Force Analysis

Within the limits of the workspace, the robot addressed on the establishment of the force field. Assuming that the inertia and friction force can be neglected, the force field generated by the elastic ropes can be calculated by the simplified Equation (12) in the HSV (hue, saturation, and value) color scale.

**Figure 7:**
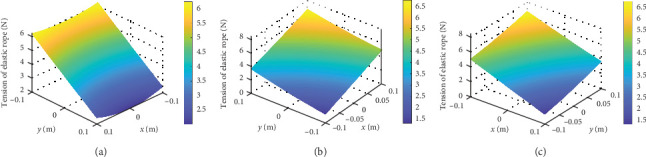
The tension of the elastic ropes with respect to the workspace. The HSV color scale encodes the tension of the elastic ropes in the space and the unit of the scale is Newton (N). The yellow color indicates areas with higher tension of the elastic rope. (a) Tension of rope 1. (b) Tension of rope 2. (c) Tension of rope 3.

**Figure 8:**
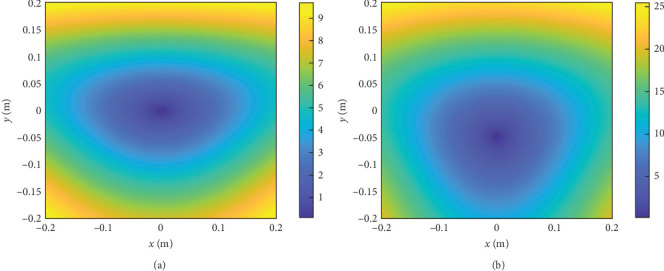
Force field generated by the elastic ropes. The HSV color scale encodes the force field in the workspace and the unit of the scale is Newton (N). Blue color indicates areas with small force value on the centre and yellow indicates areas with higher force value on the edge. (a) *k*_1_ = *k*_2_ = *k*_3_ = 20 N/m. (b) *k*_1_ = 30, *k*_2_ = 50, and *k*_3_ = 50 N/m.

Figure 7 shows the tension of the elastic ropes with respect to the workspace, the HSV color represents the value of the tension. The tension changes with the location of the robot and the minimum value of tension of rope 1 is 2 N with *x* = 0 m and *y* = 0.1 m, maximum value of tension of rope 1 is 6.1 N with *x* = ±0.1 m and *y* = −0.1 m. The tension value can be observed in Figure 7. Then, the force field within the workspace can be obtained, as shown in the Figure 8, the feedback force changes with the location of the robot. The cone-shaped force field can be observed when *k*_1_ = *k*_2_ = *k*_3_ = 20 N/m, as shown in Figure 8a, the minimum value of force is 0 N with *x* = 0 m and *y* = 0 m, maximum value of force is 9.6 N with *x* = ±0.2 m and *y* = −0.2 m. By replacing the stiffness of the elastic ropes, as shown in Figure 8b, we can change the force field to motivate the user.

Figure 8 shows the results for the static case within the workspace. The color of the dots represents the value of the force. The maximum force of the whole workspace is 9.8 N with k1 = k2 = k3 = 20 N/m, while the robot center is at the edge on the *OXY*-plane. When the robot center is far away from origin point, the feedback force is gradually increasing, so the HSV value is getting big. The minimum HSV within the whole workspace is 0 N, while the robot center is near the mass center of the table.

**Figure 9:**
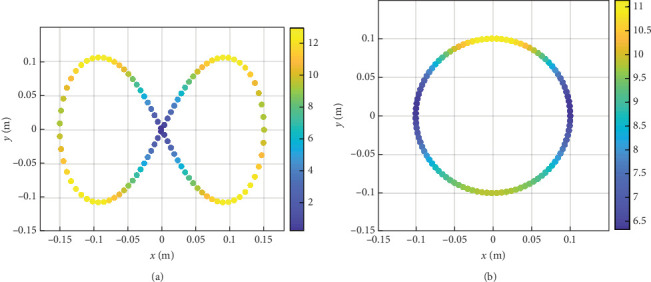
Feedback force acting on the hand during the path tracking game and the unit of the scale is Newton (N). Blue color indicates areas with small force value and yellow indicates areas with higher force. (a) “∞” path. (b) Circular path.

When the robot moves along the RT from Equations (15)–(18), the feedback force can be obtained from the force sensors and dynamic modeling. As shown in Figure 9, the feedback force varies with the location of the robot, therefore, the user can experience various assistance force to facilitate the motor learning [29, 32, 34]. Figure 9 shows the HSV results with respect to the interaction force along the “∞” and circular path. The maximum force of the “∞” path is 13.2 N and the force is ever-changing along the path. The maximum force of the circular path is 11.02 N and the minimum force is 6.48 N and the force is also everchanging along the path.

## 5. Conclusions

To address issues of the rehab training for the dyskinesia patients and cerebral palsy children, this paper proposed an arm rehab robot with OMP for upper limbs strength training. The present work studies the kinematic performance and demonstrates the capability of the arm rehab robot for force feedback. With this robot, the user's arm can move naturally within the workspace and the system can provide the position and force feedback via the sensors. The proposed training method is implemented through the kinematic and dynamic modeling. The performance analysis showed the ST and force field within the space, which is conducive to generate different force field for various patient populations. The present work has paved a way to improve the current rehab robot through direct intervention of hand position and orientation and the assistance force can be used to facilitate the motor relearning. The future work is to optimize the structure parameters and assemble the prototype for clinic trial.

## Data Availability

The simulation results and experimental results data used to support the findings of this study are available from the corresponding author upon request.

## References

[1] Feigin V. L., Stark B. A., Johnson C. O. (2021). Global, Regional, and National Burden of Stroke and Its Risk Factors, 1990-2019: A Systematic Analysis for the Global Burden of Disease Study 2019. *The Lancet Neurology*.

[2] Feigin V. L., Lawes C. M. M., Bennett D. A., Anderson C. S. (2003). Stroke Epidemiology: A Review of Population-Based Studies of Incidence, Prevalence, and Case-Fatality in the Late 20th Century. *The Lancet Neurology*.

[3] Nichols-Larsen D. S., Clark P. C., Zeringue A., Greenspan A., Blanton S. (2005). Factors Influencing Stroke Survivors’ Quality of Life During Subacute Recovery. *Stroke*.

[4] Kwakkel G., Kollen B. J., van der Grond J., Prevo A. J. H. (2003). Probability of Regaining Dexterity in the Flaccid Upper Limb: Impact of Severity of Paresis and Time Since Onset in Acute Stroke. *Stroke*.

[5] Dal’Bello L. R., Izawa J. (2021). Task-Relevant and Task-Irrelevant Variability Causally Shape Error-Based Motor Learning. *Neural Networks*.

[6] Ashburn A., Pickering R., McIntosh E. (2019). Exercise- and Strategy-Based Physiotherapy-Delivered Intervention for Preventing Repeat Falls in People With Parkinson’s: the PDSAFE RCT. *Health Technology Assessment (Winchester, England)*.

[7] Chien W.-T., Chong Y.-Y., Tse M.-K., Chien C.-W., Cheng H.-Y. (2020). Robot-Assisted Therapy for Upper-Limb Rehabilitation in Subacute Stroke Patients: A Systematic Review and Meta-Analysis. *Brain and Behavior*.

[8] Jiang Y.-C., Zheng C., Ma R. (2024). Within-Session Reliability of fNIRS in Robot-Assisted Upper-Limb Training. *IEEE Transactions on Neural Systems and Rehabilitation Engineering*.

[9] Dehem S., Gilliaux M., Stoquart G. (2019). Effectiveness of Upper-Limb Robotic-Assisted Therapy in the Early Rehabilitation Phase After Stroke: A Single-Blind, Randomised, Controlled Trial. *Annals of Physical and Rehabilitation Medicine*.

[10] Chinembiri B., Ming Z., Kai S., Xiu Fang Z., Wei C. (2021). The Fourier M2 Robotic Machine Combined With Occupational Therapy on Post-Stroke Upper Limb Function and Independence-Related Quality of Life: A Randomized Clinical Trial. *Topics in Stroke Rehabilitation*.

[11] Ting W., Aiguo S. (2019). An Adaptive Iterative Learning Based Impedance Control for Robot-Aided Upper-Limb Passive Rehabilitation. *Frontiers in Robotics and AI*.

[12] Washabaugh E. P., Guo J., Chang C.-K., Remy C. D., Krishnan C. (2019). A Portable Passive Rehabilitation Robot for Upper-Extremity Functional Resistance Training. *IEEE Transactions on Biomedical Engineering*.

[13] Zongxing L., Jie Z., Ligang Y., Jinshui C., Hongbin L. (2024). The Human-Machine Interaction Methods and Strategies for Upper and Lower Extremity Rehabilitation Robots: A Review. *IEEE Sensors Journal*.

[14] Proietti T., Guigon E., Roby-Brami A., Jarrassé N. (2017). Modifying Upper-Limb Inter-Joint Coordination in Healthy Subjects by Training With a Robotic Exoskeleton. *Journal of NeuroEngineering and Rehabilitation*.

[15] Hidler J., Nichols D., Pelliccio M., Brady K. (2005). Advances in the Understanding and Treatment of Stroke Impairment Using Robotic Devices. *Topics in Stroke Rehabilitation*.

[16] Oblak J., Matjačić Z. (2011). Design of a Series Visco-Elastic Actuator for Multi-Purpose Rehabilitation Haptic Device. *Journal of NeuroEngineering and Rehabilitation*.

[17] Krebs H. I., Ferraro M., Buerger S. P. (2004). Rehabilitation Robotics: Pilot Trial of a Spatial Extension for MIT-Manus. *Journal of NeuroEngineering and Rehabilitation*.

[18] Melendez-Calderon A., Masia L., Gassert R., Sandini G., Burdet E. (2011). Force Field Adaptation Can Be Learned Using Vision in the Absence of Proprioceptive Error. *IEEE Transactions on Neural Systems and Rehabilitation Engineering*.

[19] Furukawa J.-I., Noda T., Teramae T., Morimoto J. (2017). Human Movement Modeling to Detect Biosignal Sensor Failures for Myoelectric Assistive Robot Control. *IEEE Transactions on Robotics*.

[20] Li Z., Li X., Li Q., Su H., Kan Z., He W. (2022). Human-in-the-Loop Control of Soft Exosuits Using Impedance Learning on Different Terrains. *IEEE Transactions on Robotics*.

[21] Kong L., He W., Dong Y., Cheng L., Yang C., Li Z. (2021). Asymmetric Bounded Neural Control for An Uncertain Robot by State Feedback and Output Feedback. *IEEE Transactions on Systems, Man, and Cybernetics: Systems*.

[22] Chen Z., Wang C., Fan W. (2020). Robot-Assisted Arm Training Versus Therapist-Mediated Training After Stroke: A Systematic Review and Meta-Analysis. *Journal of Healthcare Engineering*.

[23] Lum P. S., Burgar C. G., Shor P. C., Majmundar M., Van der Loos M. (2002). Robot-Assisted Movement Training Compared With Conventional Therapy Techniques for the Rehabilitation of Upper-Limb Motor Function After Stroke. *Archives of Physical Medicine and Rehabilitation*.

[24] Basteris A., Nijenhuis S. M., Stienen A. H. A., Buurke J. H., Prange G. B., Amirabdollahian F. (2014). Training Modalities in Robot-Mediated Upper Limb Rehabilitation in Stroke: A Framework for Classification Based on a Systematic Review. *Journal of NeuroEngineering and Rehabilitation*.

[25] Mehrholz J., Pohl M., Platz T., Kugler J., Elsner B., Cochrane Stroke Group (2015). Electromechanical and Robot-Assisted Arm Training for Improving Activities of Daily Living, Arm Function, and Arm Muscle Strength After Stroke. *Cochrane Database of Systematic Reviews*.

[26] Tigrini A., Verdini F., Fioretti S., Mengarelli A. (2023). On the Decoding of Shoulder Joint Intent of Motion From Transient EMG: Feature Evaluation and Classification. *IEEE Transactions on Medical Robotics and Bionics*.

[27] Tigrini A., Mengarelli A., Cardarelli S., Fioretti S., Verdini F. (2020). Improving EMG Signal Change Point Detection for Low SNR by Using Extended Teager-Kaiser Energy Operator. *IEEE Transactions on Medical Robotics and Bionics*.

[28] Guillén-Climent S., Garzo A., Muoz-Alcaraz M. N., Casado-Adam P., Mayordomo-Riera F. J. (2021). A Usability Study in Patients With Stroke Using MERLIN, A Robotic System Based on Serious Games for Upper Limb Rehabilitation in the Home Setting. *Journal of NeuroEngineering and Rehabilitation*.

[29] Ji J. C., Wang Y., Zhang G., Lin Y., Wang G. (2021). Design and Simulation Analysis of a Robot-Assisted Gait Trainer With the PBWS System. *Journal of Healthcare Engineering*.

[30] Hunt J., Lee H., Artemiadis P. (2017). A Novel Shoulder Exoskeleton Robot Using Parallel Actuation and a Passive Slip Interface. *Journal of Mechanisms and Robotics*.

[31] Demofonti A., Carpino G., Zollo L., Johnson M. J. (2021). Affordable Robotics for Upper Limb Stroke Rehabilitation in Developing Countries: A Systematic Review. *IEEE Transactions on Medical Robotics and Bionics*.

[32] Ji J., Guo S., Xi F., Zhang L. (2020). Design and Analysis of a Smart Rehabilitation Walker With Passive Pelvic Mechanism. *Journal of Mechanisms and Robotics*.

[33] Dan Z., Xi F., Mechefske C. M., Lang S. (2004). Analysis of Parallel Kinematic Machine With Kinetostatic Modelling Method. *Robotics & Computer Integrated Manufacturing*.

[34] Guo L., Lu Z., Yao L. (2021). Human-Machine Interaction Sensing Technology Based on Hand Gesture Recognition: A Review. *IEEE Transactions on Human-Machine Systems*.

